# ANN Prediction of Laser Power, Cutting Speed, and Number of Cut Annual Rings and Their Influence on Selected Cutting Characteristics of Spruce Wood for CO_2_ Laser Processing

**DOI:** 10.3390/ma17133333

**Published:** 2024-07-05

**Authors:** Ivan Ružiak, Rastislav Igaz, Ivan Kubovský, Eugenia Mariana Tudor, Milada Gajtanska, Andrej Jankech

**Affiliations:** 1 Faculty of Wood Sciences and Technology, Department of Physics, Electrical Engineering and Applied Mechanics, Technical University in Zvolen, T. G. Masaryka 24, 96001 Zvolen, Slovakia; igaz@tuzvo.sk (R.I.); kubovsky@tuzvo.sk (I.K.); 2 Green Engineering and Circular Design Department, Salzburg University of Applied Sciences, Markt 136a, 5431 Kuchl, Austria; eugenia.tudor@fh-salzburg.ac.at; 3 Faculty of Furniture Design and Wood Engineering, Transilvania University of Brasov, B-dul. Eroilor nr. 29, 500036 Brasov, Romania; 4 Faculty of Wood Sciences and Technology, Department of Mathematics and Descriptive Geometry, Technical University in Zvolen, T. G. Masaryka 24, 96001 Zvolen, Slovakia; gajtanska@tuzvo.sk (M.G.); jankech@tuzvo.sk (A.J.)

**Keywords:** CO_2_ laser, artificial neural networks, spruce wood, cutting kerf, heat-affected zone, sensitivity analysis

## Abstract

In this work, we focus on the prediction of the influence of CO_2_ laser parameters on the kerf properties of cut spruce wood. Laser kerf cutting is mainly characterized by the width of kerf and the width of the heat-affected zone, which depend on the laser power, cutting speed, and structure of the cut wood, represented by the number of cut annual rings. According to the measurement results and ANN prediction results, for lower values of the laser power (*P*) and cutting speed (*v*), the effect of annual rings (ARs) is non-negligible. The results of the sensitivity analysis show that the effect of *v* increases at higher energy density (*E*) values. With *P* in the range between 100 and 500 W, *v* values between 3 and 50 mm·s^−1^, and AR numbers between 3 and 11, the combination of *P* = 200 W and *v* = 50 mm·s^−1^, regardless of the AR value, leads to the best cut quality for spruce wood. In this paper, the main goal is to show how changes in the input parameters affect the characteristics of the cutting kerf and heat-affected zones for all possible input parameter values.

## 1. Introduction

Laser wood cutting is a standard method for processing wood, and powerful CO_2_ lasers are used for this purpose. CO_2_ lasers have been used for decades and achieve a high-quality cut with a wide range of material thicknesses. In a wide range of cases, CO_2_ laser cutting is preferred over water-jet cutting. A good-quality cut surface is crucial for the subsequent processing steps. Another advantage of laser cutting lies in the fact that the laser affects only a limited area via thermal stress, and, in contrast to water jets, it does not affect the sample’s moisture content [1,2,3,4,5,6].

**Figure 1:**
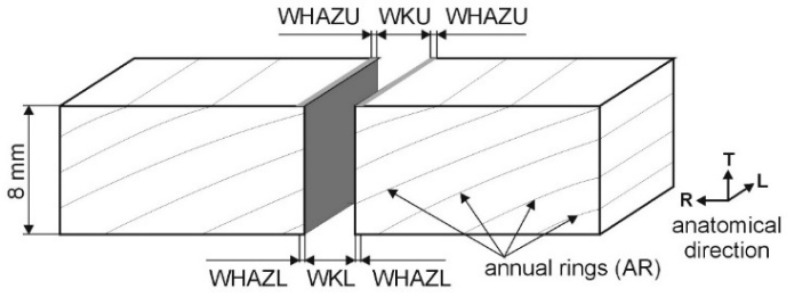
CO_2_ laser cutting scheme for a wood sample.

The properties of the cut are important for the usage of the sample because they significantly affect the wood’s surface properties, such as roughness, and the ability to prepare glued composites is mainly controlled by the strength of the glue joint. Therefore, it is important to optimize the cut quality according to the changes in the cut parameters vs. the laser power (*P*), cutting speed (*v*), density profiles, and many more. One of the main criteria of a good-quality cut is that the cutting kerf width and thickness profile do not significantly change. This can be characterized by the ratios of the cutting kerf widths on the primary cut side on the sample and on the opposite side. To optimize the cut quality, Eltawahni et al. [7] defined a methodology for the evaluation of the characteristic WKR, defined as the ratio of the cutting kerf width on the lower surface (WKL) divided by the cutting kerf width on the upper surface (WKU) (WKR = WKL/WKU) (Figure 1), which is mainly affected by *P*, *v*, and the position of the focal point. In another study, Eltawahni et al. [8] studied the effects of the laser characteristics on the cutting parameters of plywood materials.

Kubovský et al. studied the effects of parameters *v*, the number of cut ARs, and *P* on the cut characteristics of spruce wood for measured values [9]. The study also included an ANOVA to show the combined effect of all three input parameters on each output parameter, but only for low *P* (max.: 150 W) and *v* (max.: 9 mm∙s^−1^) values.

When cutting spruce wood with a CO_2_ laser, the laser output power is usually up to about 500 W, and the cutting speed does not exceed 50 mm∙s^−1^. The number of cut ARs for spruce wood is typically from 3 to 11. In order to determine the influence of the *P* and AR parameters, Ružiak et al. [10] used artificial neural networks (ANNs), keeping the cutting speed constant (*v* = 12 mm·s^−1^), to predict the WKU, WKL, WKR, WHAZU, and WHAZL parameters (Figure 1). 

Many other authors have conducted similar research using other wood-based materials. Nukman et al. [11] studied the effect of these technological parameters on the cut quality for Malaysian-based wood and plywood. They presented the dependence of the material removal rate (MRR) vs. *P* and *v*. The MRR parameter increases with *P* in the exponential stabilizing form for various atmospheres. 

The effects of the CO_2_ laser parameters on the width of the heat-affected zone in wood and wood composites was studied in [12].

There are many articles that deal with the laser processing of metallic or polymer materials via CO_2_ laser. However, only a few authors have studied the effect of the laser characteristics on the cut quality for wood materials. 

The following paragraph is mainly focused on the influence of the *v* and *P* values on the width and the resulting cut quality.

Martinez-Conde et al. [4] are some of the few authors who have studied the effects of CO_2_ laser parameters on the cut quality of wooden materials. They compared the results of the cut characteristics via CO_2_ laser vs. other conventional lumber-cutting techniques. They also studied the effects of laser characteristics on the cutting kerf width of wood. Similarly to other authors, they found that *P* increased the cutting kerf width and that *v* decreased it nonlinearly.

Lum et al. found the same effect of *v* on the cutting kerf width for medium-density fiberboard (MDF) [13]. However, there have been few studies dealing with the effects of laser characteristics on the cutting kerf width of the final product compared to the cutting of metals and plastics. Authors dealing with the effects of *v* on cutting kerf widths have determined that there is a nonlinear decrease in the cutting kerf width with *v*, as shown in [14,15].

The authors of [16] studied how UV laser characteristics affect the cutting parameters of Japanese larch, cedar, and beech wood. In another study [17], the authors compared UV–VIS–NIR lasers for their abilities to cut selected species of wood. 

Artificial neural networks (ANNs) have often been applied in material science and in the optimization of technologies for materials. Many authors have used ANNs to predict material properties. The authors of [18] predicted the thermal conductivity of wood. The surface roughness of wood during machining was predicted in [19], and an ANN was applied for the prediction of the surface roughness and energy consumption of the wood in [20]. The ANNs in [21] were able to predict the optimal power consumption in wood processing.

ANNs have also been successfully applied for the optimization of technologies. The authors of [22] predicted the formaldehyde emissions of particleboard from the manufacturing conditions. The authors of [23] also determined the effect of the manufacturing process characteristics on the modulus of rupture (MOR) and modulus of elasticity (MOE).

The authors of [24,25,26] predicted the bonding quality versus the manufacturing conditions using ANNs and multiple regression models. 

The optimization of the CNC process for the best wood surface quality using the ANN approach was published in [27]. In [28], the authors studied the effect of CNC processing characteristics on the surface properties of MDF using the ANN approach.

The main goal of this study was to predict the parameters of the kerf region (WKU, WKL, WKR) and heat-affected zone region (WHAZU, WHAZL) depending on the laser power (*P*) and cutting speed (*v*) at any number of cut annual rings (ARs) by means of ANNs for all possible values of all three input parameters. Another goal was to determine how each input parameter influences the cut characteristic change with increased *P* and *v* values, which was obtained from a sensitivity analysis. The results of the analysis will help us find the optimal values of *P* and *v* to achieve the best possible cut quality when using different CO_2_ laser parameters. The obtained cut characteristic trends vs. the input parameters are fully applicable for any spruce wood cut by a CO_2_ laser and quantitatively for the same thickness of spruce wood cut in a direction parallel to the wood fibers. The results of the sensitivity analysis, together with the input parameter optimization, can be used as a tool for improving the spruce wood CO_2_ laser cutting process from the first phase of *P* and *v* determination up to the final best-quality spruce wood cut.

## 2. Materials and Methods

The experiments were carried out on spruce wood (*Picea abies (L) H. Karst*). Experimental laser equipment LCS 400 (VEB Feinmechanische Werke, Halle, Germany) with maximum power output 400 W at wavelength 10.6 μm was used for cutting. The cutting kerf was obtained in the tangential direction on a sample with dimensions *T* × *R* × *L* (8 × 100 × 1000) mm with a density of *ρ* = 428.4 ± 27.9 kg·m^−3^. The samples were cut continuously using laser powers of 100 and 150 W and three cutting speeds of 3, 6, and 9 mm·s^−1^, which were selected according to the sample thickness and the limits of the laser. The focal length was 127 mm (5″), the prefocus beam diameter was 10 mm, and the spot diameter (*d*) (in Equation (1)) was 0.3 mm. The focal-point position of the laser beam was at one-half of the sample thickness. The process gas was supplied via a Laval contour nozzle with an air pressure of 0.25 MPa. The cut spruce wood samples were climatized at a temperature (*t*) of 20 °C and a relative humidity (*RH*) of 65%, which corresponded to an equilibrium moisture content (*w*) of 12 ± 1%.

The abbreviations used in the text are as follows: WKU: cutting kerf width on the upper surface; WKL: cutting kerf width on the lower surface; WKR: ratio of WKU and WKL; WHAZU: width of the heat-affected zone on the upper surface; WHAZL: width of the heat-affected zone on the lower surface; *P*: laser power; *v*: cutting speed; AR: number of cut annual rings.

The number of cut ARs was used as an input parameter because it is not possible to define the number of cut ARs per meter, as this parameter varies for each annual ring. All the research was performed only in the direction parallel to the wood fibers because it is not possible to track the number of cut ARs in the cutting kerf, as it changes on each ring.

Based on preliminary experiments, we found that if the wood sample as a cut material is placed on a standard base (steel grid), a structure that copies the base grid is created on the underside of the sample. Therefore, in the following experiments, the samples were supported during the cutting so that the lower surface was not in contact with the grid. Under the sample, a 20 mm thick space formed by air was created to minimize the influence of the lower edge of the cut due to the dispersion of hot air on the underlying grid.

All the studied cut parameters of the sample were determined from a microscope picture using K-cluster analysis, described in detail in [9,10]. The heat-affected zone in the microscope picture is the area in the darker color (with no material removal) symmetrically surrounding the cutting kerf (with material removal), as shown in Figure 1, which includes the definitions of the investigated parameters.

In our experiment, the laser beam focal length, beam diameter, spot diameter, focal-point position, and pressure were constant for all the measurements; thus, the parameters of the cut region and heat-affected zone could not be predicted. 

The applied laser device uses a commercially produced CO_2_ laser fully closed tube with a 10.6 µm wavelength as the radiation source, for which the manufacturer declares the TEM00 mode and a Gaussian distribution of the radiation intensity in the beam cross-section. We used a CO_2_ laser with an output power (*P*) of 400 W with the cutting mode of the measurement with a resolution of approximately 300 dpi.

The energy density (*E*) (J·m^−2^) with the applied *P*, *v*, and spot diameter (*d*) values given to the sample section is defined by Equation (1):

(1) $$E=\frac{P}{v.d}$$

The energy density parameter describes the dosage given to the sample and directly affects all the studied cut characteristics. According to this definition, with a constant *v*, there is a linear increase in the energy given to the material with a change in *P*. In contrast, if *P* is constant, the change in the energy density vs. *v* is hyperbolic and it decreases with increasing cutting speed.

Based on the measured values of the cut characteristics shown in Figure 1 vs. the *P*, *v*, and AR values used in the measurements (listed in the first paragraph of this section), we used an ANN to predict the change in the cut characteristics at higher values of *P* and *v.* The validity of such an approach is typically controlled by the coefficient of determination (R^2^) and root-mean-square error (RMSE).

The processing of the measured data using the ANN method was completely performed in the Statistica 12.0 program (StatSoft (Europe) GmbH, Hamburg, Germany). Based on the input conditions and taking into account the error minimization between the measured and predicted data, the software found the optimal neural network. The MLP 3-3-5 multilayer perceptron neural network with error backpropagation was found to be the best neural network for the prediction of all the output parameters. The network input consisted of three neurons (one for the laser power (*P*), one for the cutting speed (*v*), and one for the number of cut ARs), the output consisted of five neurons (for the WKU, WKL, WKR, WHAZU, and WHAZL), and the number of hidden neurons was equal to three at level one. Statistica 12.0 software was also used to analyze the sensitivity of the neural network and calculate the determination coefficient (R^2^) and the relative root mean square error (Rel_RMSE).

The R^2^ (computed as the square of the correlation coefficient) should be as close as possible to 1. The second statistical parameter, the RMSE, is the average value of the sum of squares between the measured and predicted values. This parameter should be as minimal as possible, but this depends on the average value of the given parameter. Therefore, it is standard practice to replace this parameter with the relative root mean square error (Rel_RMSE) value, which is a universal statistical parameter defined as the ratio of the RMSE to the average parameter value obtained via the measurement. This value should be close to 0.

The ideal combination of these two parameters is R^2^ = 1 and Rel_RMSE = 0, but these values can only be obtained if all the predicted values are the same as the measured ones, which is not possible in praxis.

Therefore, the standard method is to compare the Rel_RMSE parameter with the property measurement error vs. the 95% confidence interval error (equal to 0.05). If a prediction model has a Rel_RMSE value lower than the measurement or statistical error, then the model is valid and can be successfully applied for the prediction of new nonmeasured input parameter values. 

The R^2^ parameter is sensitive to measurement error. The measurement uncertainty is low for homogenous materials; thus, R^2^ values higher than 0.99 are considered valid. This applies for metallic materials and some polymeric materials.

As material is less homogenous or anisotropic as the measurement uncertainty increases, lower R^2^ values are obtained; thus, R^2^ values above 0.9 are also considered very good. 

The R^2^ and Rel_RMSE parameters are used the same for the ANN prediction and mathematical regression models. The standard fitting procedures take all the input data as one group to look for mathematical equations between the output and input parameters.

The difference in ANNs vs. standard fitting procedures lies in the fact that ANNs divide the measured data into three independent groups: training, testing, and validation. These three groups are each based on different data; thus, all the studied input parameters change for one group vs. another. The neural network used these three groups to compute the network performance and error. If the network error does not significantly change between groups, then the change in the input parameters does not affect the neural network statistics. 

Another difference between these two methods is that the ANN prediction output is not a mathematical equation but rather the output values vs. the input values with new input parameter combinations.

## 3. Results

We divided the results and discussion into two basic sections. The first section illustrates the results of the cut characteristics vs. AR at *P* values of 100 and 150 W and *v* values of 3, 6, and 9 mm·s^−1^. The second section deals with the optimization of the cutting characteristics at *P* values between 100 W and 500 W, *v* values between 3 and 50 mm·s^−1^, and numbers of cut ARs between 3 and 11. All the results are discussed in the context of references, were statistically processed, and are discussed again according to the energy density values because these have a direct effect on the creation of the cutting kerf width and the width of the heat-affected zone.

As mentioned above, the energy density is defined by Equation (1), where the parameters *P* and *v* were changed for each measurement and prediction. The energy density values vs. the laser power (*P*) and cutting speed (*v*) are shown in Table 1. The *E* values marked in red are measurements, and the values marked in black are predictions.

### 3.1. Measured Values

The effect of ARs on all the studied cut characteristics for low *P* values was statistically significant according to 95% confidence intervals. However, in comparison with the *P* and *v* effects, the effect of ARs was very small, as only *P* and *v* affect the energy density, according to Equation (1) [10].

The following notation is used in this section. The acronyms (e.g., WKU) refer to the output parameters in the figure, and the subsequent numbers specify the *P* or *v* value. For example, WKU100 denotes the cutting kerf on the upper board for *P* equal to 100 W, and WKR9 corresponds to the cutting kerf ratio (WKR) at a *v* of 9 mm·s^−1^.

**Figure 2:**
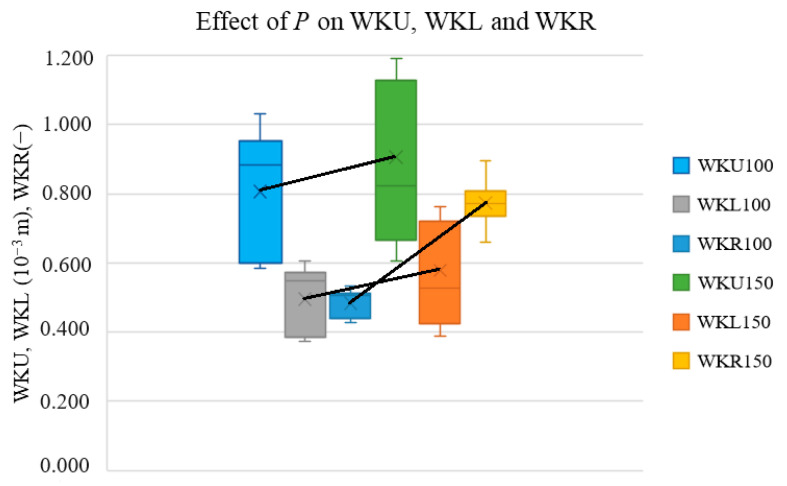
Effects of *P* on cutting kerf widths and their ratios.

**Figure 3:**
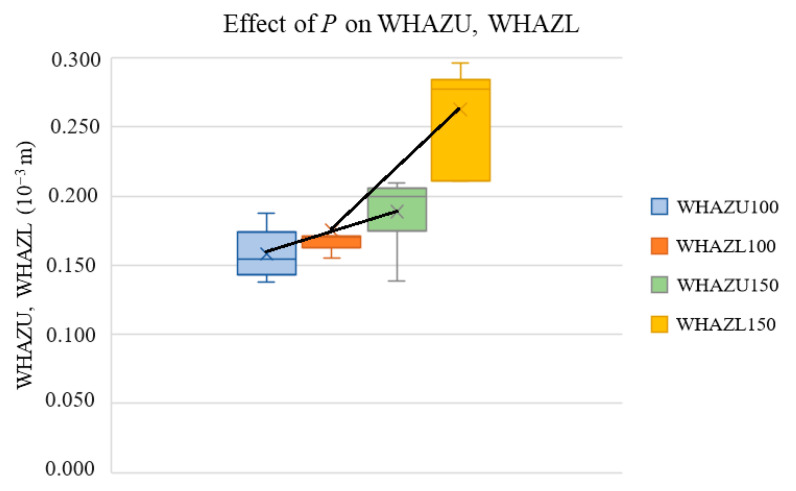
Effects of *P* on widths of heat-affected zones.

**Figure 4:**
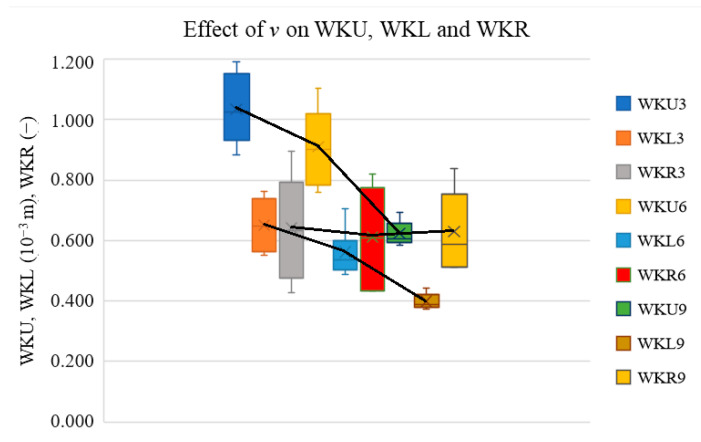
Effect of *v* on cutting kerf widths and their ratios.

**Figure 5:**
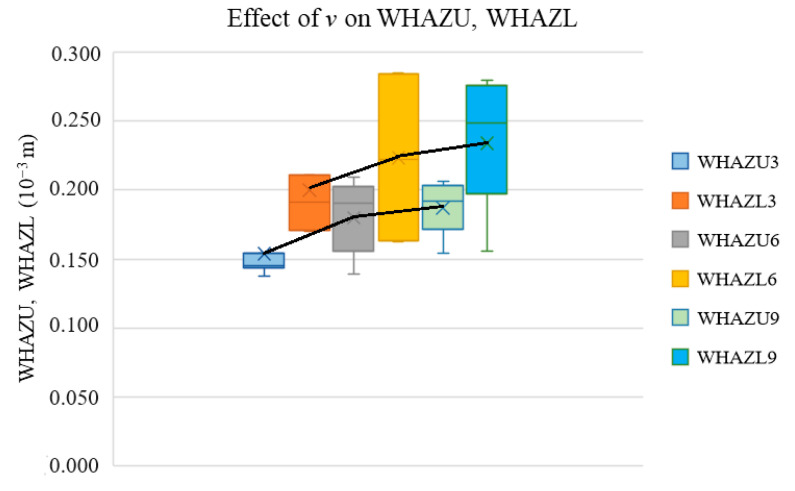
Effect of *v* on widths of heat-affected zones.

In this section, we present box–whisker plots not only to show the trends of the selected cut characteristics vs. *P* and *v* but also to show whether the change in the output parameters was statistically significant. In this setup, the distance from the middle point to the top of the bar is equal to the standard deviation of the measurement. If the trend change is significantly lower than this distance, then this change cannot be considered significant. Thus, it is not possible to conclude that the input parameter affects the output parameter. Figure 2, Figure 3, Figure 4 and Figure 5 present the effect of *P* or *v* on the cutting parameters obtained via measurement (red *E* values in Table 1).

Figure 2 presents the effect of *P* on the measured WKU, WKL, and WKR values. The letter nomenclature is the same as in Section 2, and the numbers 100 and 150 correspond to the values at *P* = 100 W and 150 W, respectively.

The graph shows that *P* increased the WKU and WKL values and that the WKR increased considerably with the change in *P* from 100 to 150 W (light-blue bar at 100 W and yellow bar at 150 W). For lower *P* values (maximum to 150 W), the slope of WKR vs. *P* is by far the highest; thus, *P* had the most significant effect on WKR among all the parameters of the cutting kerf region. The maximum average kerf width value is approx. 0.75, which is still a low value for a good cut quality. Therefore, to achieve a better cut quality, a higher *P* value must be used.

Increasing *P* by maintaining the *v* and d values leads to an increase in the energy density (*E*) given to the sample, which increases the heat given to the material and, thus, also the cutting kerf width. 

Figure 3 presents the effect of *P* on the measured widths of the heat-affected zones (WHAZU, WHAZL).

The graph shows that the *P* increases the width values of the heat-affected zones. This is due to the fact that, at higher *P* values, the total heat given to the sample is higher, which increases both the heat needed for the evaporation of the material (creation of cutting kerf) and that needed for the creation of the heat-affected zone. 

In addition, it also shows that the WHAZL values are higher than the WHAZU values because, when the laser cuts the lower board, the hot air generated by cutting flows out of the cutting kerf region and, thus, mainly affects the heat-affected zone at the expense of decreasing the cutting kerf width at the lower surface. 

Figure 2 and Figure 3 also show that the *P* had a more significant effect on the WHAZL than on the WKL, which can be attributed to the fact that, after cutting through the sample, hot air is generated by the cutting flows outside of the cutting kerf, decreasing the WKL. A higher *P* value increases the density of the energy that is used on the lower board mainly in the heat transfer to the heat-affected zone, because the hot air does not remain in the cutting kerf region.

Figure 4 presents the effect of *v* on the measured WKU, WKL, and WKR values. The letter nomenclature is the same as in Section 2, and the numbers 3, 6, and 9 correspond to *v* = 3, 6, and 9 mm·s^−1^, respectively.

The graph shows that the *v* increase resulted in a significant decrease in the cutting kerf widths on the upper and lower boards but did not have a significant effect on their ratios, which is because, with the increase in the *v*, the *E* values linearly decreased, and thus, the heat given to the material decreased, as well as the cutting kerf values.

The graph also shows that the WKU is higher than the WKL. During wood cutting, air in the cutting kerf occurs. As the laser beam continues to the lower surface, more air is generated in the cutting kerf. This air is heated by the heat transferred to the material through the energy density and that flows out of the cutting kerf, which causes higher heat losses in the cutting kerf region, thereby decreasing the WKL vs. WKU. This effect is more significant for lower *v* values because, at higher *v* values, the heat losses that result from hot air flowing out are higher.

Figure 5 presents the effect of *v* on the measured widths of the WHAZU and WHAZL of the heat-affected zones.

The graph illustrates that, within a *v* range lower than 6 mm·s^−1^, the WHAZU and WHAZL values increased, although the differences in the WHAZU and WHAZL at *v* = 6 and 9 mm·s^−1^ are not statistically significant. 

As the laser beam cuts through the material, the heat transfer via the flow of hot air to the heat-affected zone increases, resulting in an increase in the width of the heat-affected zone at the expense of a decrease in the cutting kerf width. The heat transfer from the cutting kerf region to the heat-affected zone is significant mainly for low energy density (*E*) values. This increase is reduced only to a certain *v* value because, at high *v* values, the density of the energy (*E*) given to the sample is low, which also decreases the effect of the heat transfer losses to the heat-affected zone. Nevertheless, versus the standard deviation of the measurement (one-half of the bar height), this increase cannot be considered statistically significant for *v* values between 6 and 9 mm·s^−1^.

### 3.2. Comparison of ANN Approach and Mathematical Regression Models

The data obtained from the measurements were statistically processed via the ANN and nonlinear mathematical regression. We needed to choose the best approach from these different points of view, which was performed mainly via a comparison of the statistical parameters between the predicted and real measured data considering both points of view. The R^2^ values are valid for laser power (*P*) values of 100 and 150 W, cutting-speed (*v*) values of 3, 6, and 9 mm·s^−1^, and for all possible values of the number of ARs, because it is only at these input values that the cut parameters could be measured and compared.

The authors of [9] present the results of the predicted cut characteristics vs. all the studied input parameters, the determination coefficients (R^2^) of which are listed in Table 2.

The authors of [10] present the results of the predicted cut characteristics vs. all the studied input parameters, the determination coefficients (R^2^) of which are listed in Table 3.

According to a comparison of both tables, the ANN approach was significantly better at predicting the measured data. The MLP 3-3-5 multilayer perceptron network with the backpropagation error algorithm proved to be the best neural network for the prediction of all the studied cut characteristics, the results of which are presented in the next sections. 

The statistical parameter results show that the correlation coefficient was minimal at 0.966 and the relative root mean square error was maximal at 3.5% for the WKR (which is higher because it is bound to the WKU and WKL values). These values for the wood-based material are almost at the upper bound mainly because of the material’s anisotropy, inhomogeneity, and nonuniform moisture content distribution, which cannot be measured, and therefore, their effects on the studied cut characteristics cannot be predicted. In general, it can be said that the coefficient of correlation and root-mean-square error can be improved only by taking into consideration the other laser parameters mentioned in Section 2. However, these parameters, according to the references, do not have as strong an effect on the cut characteristics as the *P* and *v.*

### 3.3. ANN Prediction

In the experimental design, the input values used to train the ANN were *P* values of 100 and 150 W, a number-of-cut-AR range from 3 to 11, and *v* values of 3, 6, and 9 mm·s^−1^; thus, the training group had 54 lines. The validity of the model is dependent only on the number of input data combinations and is not affected by the number of values for each input parameter (the *P*, *v*, and AR). According to the very high R^2^ parameter values for the ANN prediction model versus the variability in the wooden-material properties, the ANN prediction model is valid. We obtained five artificial neural networks for the prediction of the WKU, WHAZU, WKL, WHAZL, and WKR with the best statistical parameters. In the prediction, we used the MLP and RBF networks. The activation functions used for the hidden-layer neurons were identity, logistic, atanh, exponential, and sinus. The activation functions used for the output neurons were the same as those used for the hidden layer. The numbers of hidden neurons for both the MLP and RBF networks were of the maximum possible intervals, meaning that the number of hidden neurons for the MLP network was between 1 and 54 and the number of those for the RBF network was between 1 and 38.

The measurement data were divided into a training group, testing group, and validation group at a ratio of 70%:15%:15%. The validity of the ANN prediction model was controlled by the determination coefficients (R^2^) between the prediction and measurement values, relative root mean square error (Rel_RMSE), and error propagation, which were published in [10]. The coefficient of correlation between the predicted and measured values was minimal at 0.966 for the ratio of the cutting kerf widths (which is not an independent parameter) and increased to the maximum value of 0.99. In addition, the relative root mean square error was between 1 and 2% for the widths of the cutting kerf and the widths of the heat-affected zones (those that were measured). Therefore, in general, these statistical parameters are at their upper bounds for wood-based materials, which, even in a steady state, are not homogeneous, are anisotropic, and even show nonuniform moisture content distribution. The thermal degradation caused by the CO_2_ laser can even increase the uncertainty of the measurement or prediction.

If the overall statistics are at high levels, then neural networks can predict the output values even with changed input values. According to the neural network statistics published in [10], the network error is very low and does not show any strong changes between groups (a 1% error in training, a 1.3% error for testing, and a 1.1% error for validation) vs. the measurement error, which is from approximately 15% to 20% of the variance coefficient level. Furthermore, the R^2^ and Rel_RMSE values are at a very high level for wood-based materials; thus, the MLP 3-3-5 neural network with the backpropagation error can predict all the studied cut characteristics vs. the *P*, *v*, and number of ARs.

The WKR parameter can be defined for any *P*, *v*, and number-of-AR values with the equation WKR = WKL/WKU. Values for the measuring subset were computed from the measured WKU and WKL values with the same combination of all three input parameters. The theoretical predictions of the WKR values can be obtained in two ways: (1) by finding the predicted WKU and WKL values, and (2) by computing the WKR from the definition of the WKR and by using the WKR values with the measuring subset and predicting them vs. the *P*, *v*, and AR values.

In the data-processing phase, we applied both methods. The correlation between both approaches in terms of the predicted values for the best network is 0.93 with a root mean square error of 0.01 (which corresponds to a percentage difference of 1.43%). In this study, we used the approach for the prediction of new WKR values from the former WKR values (which were predicted, not computed) via the ANN. Even small differences between the two approaches suggest that they are both applicable.

In this article, we present the effects of *P*, AR, and *v* on the cut characteristics of spruce wood for all the possible CO_2_ laser parameters, from which it is possible to predict the cut properties of sawn spruce wood at any *P* between 100 and 500 W, any AR between 3 and 11, and any *v* between 3 and 50 mm.s^−1^ with the goal of optimizing the cutting process covering all possible technological parameter combinations at which spruce wood can be cut.

**Figure 6:**
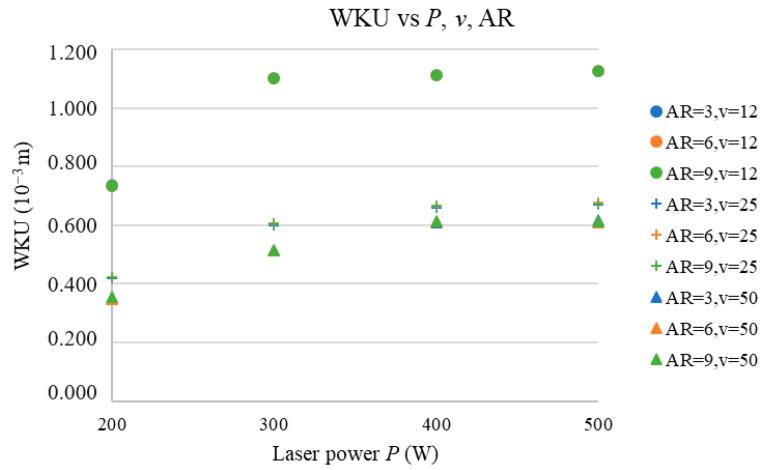
WKU vs. *P*, *v*, and AR.

**Figure 7:**
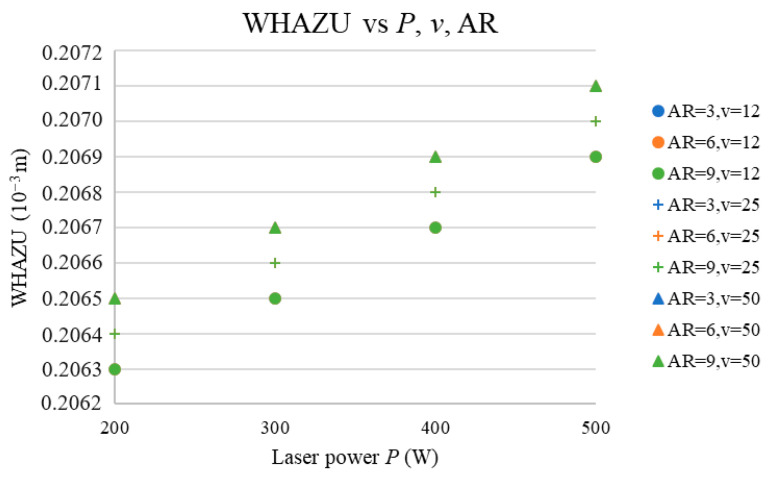
WHAZU vs. *P*, *v*, and AR.

**Figure 8:**
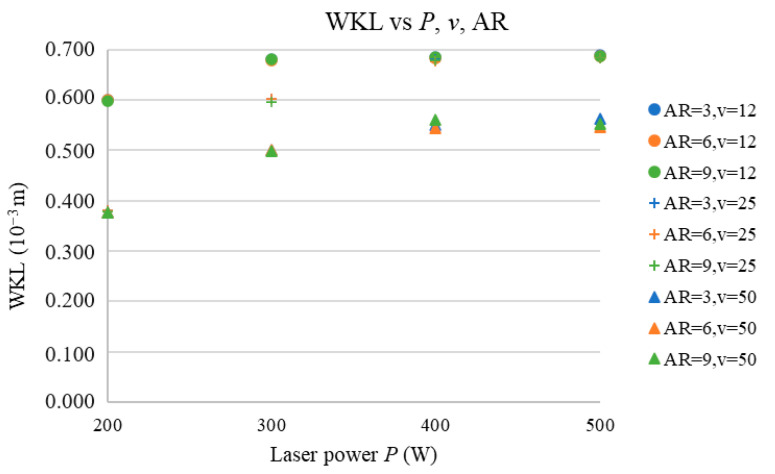
WKL vs. *P*, *v*, and AR.

**Figure 9:**
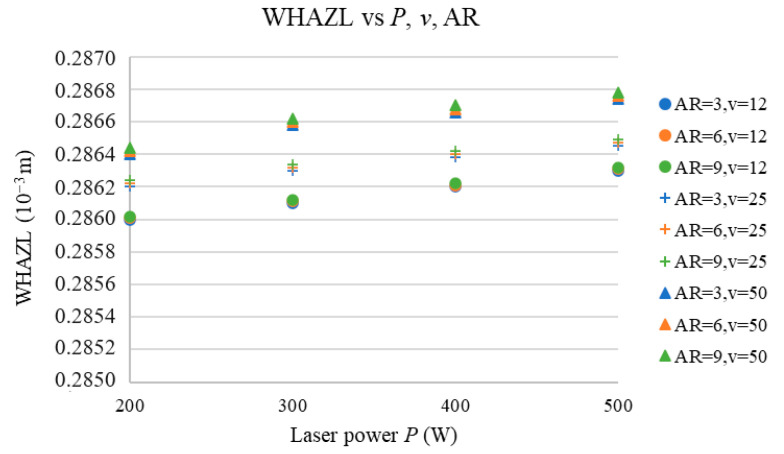
WHAZL vs. *P*, *v*, and AR.

**Figure 10:**
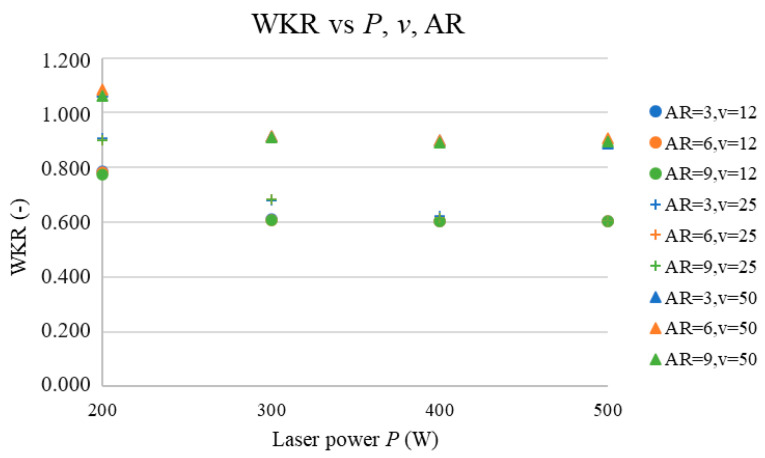
WKR vs. *P*, *v*, and AR.

The next three subsections address the prediction of the (WKU, WHAZU), (WKL, WHAZL), and WKR vs. the *P*, AR, and *v* and discuss the effects of *P* and AR on all the predicted parameters. Figure 6, Figure 7, Figure 8, Figure 9 and Figure 10 show the predicted values of the cut characteristics at nonmeasured *P* and *v* values by maintaining the same AR values. Predictions were made for laser power (*P*) values of 200, 300, …, 500 W and cutting-speed (*v*) values of 12, 25, and 50 mm·s^−1^; thus, they were higher than the values used in the measurement. Thus, it is not possible to combine the results in Figure 2, Figure 3, Figure 4 and Figure 5 with those presented in Figure 6, Figure 7, Figure 8, Figure 9 and Figure 10. Figure 2, Figure 3, Figure 4 and Figure 5 show the trends of the measured values, and Figure 6, Figure 7, Figure 8, Figure 9 and Figure 10 show the trends of the predicted values. According to the abovementioned ANN network statistical parameters, MLP 3-3-5 is the best neural network, as it is possible to predict the cutting parameter values with the prediction subset.

### 3.4. Prediction of WKU and WHAZU versus AR, P, and v Parameters

In this section, we present the predicted WKU and WHAZU vs. the AR, *P*, and *v*, which provide information on the quality of the spruce wood cut on the upper board. The results are presented as a graph of the output characteristics vs. the number of ARs and the *P* at three different *v* values (12, 25, and 50 mm·s^−1^).

In Figure 6, the dependence of the WKU on the AR and *P* is shown at the selected *v* values.

From Figure 6, it is clear that the number of cut ARs does not have any significant effect on the WKU for all *v* values between 12 and 50 mm·s^−1^, which is in accordance with the findings in [6]. 

It can be observed that WKU vs. *P* is an exponentially stabilizing function of the *P*, which is consistent with the fact that the material removal rate (MRR) also increases exponentially with an increase in the *P*, which was also found by the authors of [5,10,11]. The same trend was acknowledged for all the selected *v* values. This is consistent with the fact that an increase in the *P* causes an increase in the energy density (*E*) and thereby increases the WKU.

The third studied input parameter was the *v*. According to Figure 6, an increase in the *v* decreases the WKU values nonlinearly (this can be seen from the vertical gap between the results at different *v* values); thus, the highest values are obtained for the lowest *v* value. This is in accordance with other research studies [4,13,14,15] and can be explained by the fact that, at higher *v* values, the *E* value decreases, which decreases the WKU. Figure 7 presents the dependence of the WHAZU on the AR and *P* at the selected *v* values.

Figure 7 shows the effects of *P*, *v*, and number of cut AR on the WHAZU. 

Figure 7 clearly shows that, with an increase in the *P* (from left to right), the WHAZU increased, and the increase was approx. 0.3%. This increase is not statistically significant. Figure 7 also clearly shows that, with an increase in the *v* from 12 to 50 mm·s^−1^, the increase is approx. 0.05%, which is also not statistically significant. This is caused by the fact that, at higher *v* values, the energy density is very low and, thus, a very low amount of heat is transferred to the heat-affected zone, which leads to higher increases in the WHAZU and WHAZL, as mentioned.

The values of the heat-affected zone width are also not influenced by the *P* because increasing it directly increases the energy density of the laser, which causes an increase in the cutting kerf width, as shown in Figure 6. These findings are consistent with those of other research studies [6,9,10,12,13].

### 3.5. Prediction of WKL and WHAZL versus P, v, and Number of Cut ARs

This subchapter presents the predicted values of the WKL and WHAZL vs. the AR, *P*, and *v*, which provide information about the quality of sawn spruce wood cut on the lower board. The results are illustrated in graphs of the output properties vs. the AR and *P* at three different values of *v* (12, 25, and 50 mm·s^−1^).

Figure 8 presents the dependence of the WKL on the AR and *P* at the selected *v* values.

The results in Figure 8 indicate that the number of cut ARs for *v* values between 12 and 50 mm·s^−1^ does not have any significant effect on the WKL due to the fact that this parameter mainly affects the WHAZL. 

The results show that WKL vs. *P* is an exponentially stabilizing function of the *P*, which is consistent with the fact that the material removal rate (MRR) also increases exponentially with the *P*, which was recorded by the authors of [5,8,10,11,13]. The increase in the WKL vs. *P* is consistent with the fact that the *P* increases the *E* values (Table 1); thus, heat is also transferred to the material, which results in a WKL increase.

The third studied input parameter was the *v*. Figure 8 shows that increasing the *v* lowered the values of the WKL nonlinearly; thus, the highest values were obtained at the lowest *v* value, which is because the increase in the *v* decreases the *E* (Table 1), thereby reducing the WKL. 

Figure 9 presents the dependence of the WHAZL on the AR and *P* at the selected *v* values.

Figure 9 shows that the number of cut ARs (for *v* values between 12 and 50 mm·s^−1^) did not significantly affect the WHAZL values, which is because, at high cutting speeds (*v*), the density of the energy (*E*) transferred to the sample is low and thus the heat transferred to the heat-affected zone is also lower. 

Values of heat-affected zone width are slightly affected by the *P* and *v* because, at higher *P* and *v* values, the thermal energy transferred to the sample is higher and thus so is the heat transfer from the cut region. This results in an increase in the WHAZL vs. the *P* and *v*. However, the effects of *v* and *P* are not statistically significant.

### 3.6. Comparison of P, AR, and v Effects on WKU (WHAZU) vs. WKL (WHAZL)

From Figure 6, Figure 7, Figure 8 and Figure 9, the following can be concluded:

The values of the WKU are higher than the values of the WKL due to the fact that, on the lower board, the heat transfer to the heat-affected zone was more significant. 

For this reason, the WHAZL values are higher than those of the WHAZU. Changes in the WHAZL are bound to changes in the WKL. More heat flowing out of the cutting kerf at a lower surface causes more heat transfer to the heat-affected zone and thus increases the WHAZL. A similar effect is also visible for the upper board, as it is only at the upper board that the creation of WKU dominates over the creation of the WHAZU.

The smallest difference in the WKU and WKL values was at *v* = 50 mm·s^−1^, and thus, a higher *v* value contributes to a better-quality cut. This is crucial for the other surface properties studied in sawn spruce wood. The number of ARs and the *P* and *v* do not play significant roles in either the WHAZU or the WHAZL.

### 3.7. Prediction of WKR vs. AR, P, and v

This subchapter presents the predicted cutting kerf width ratio vs. the number of cut ARs, laser power (*P*), and cutting speed (*v*) at input parameter values higher than those applied at the measurement, which provides information about the cut quality of the sawn spruce wood in the whole sample. The results are presented as a graph of the WKR vs. the AR and *P* with three different *v* values: 12, 25, and 50 mm·s^−1^. 

Figure 10 depicts the dependence of the WKR on the AR and *P* at the selected *v* values.

Figure 10 shows that the number of cut ARs does not have a significant effect on the WKR because, at higher energy density (*E*) values (black values vs. red values in Table 1), the AR effect decreases. Moreover, the laser power (*P*) has a significant effect on the WKR because changes the values of WKU and WKL. The effect of *P* on the WKR decreases with an increase in the *P* values, which is because, at laser powers higher than 200 W, both the WKU and WKL stabilize at constant values and thus their ratio limits are also constant values. The same effect can also be seen for the cutting speed (*v*), which is because an increased cutting speed (*v*) value decreases the change in the energy density (*E*), thus also reducing the effect of *v* on the cutting kerf width ratio (WKR). Figure 10 shows that the WKR values increase with an increase in the cutting-speed (*v*) values.

### 3.8. Full-Scale Optimization of Cut Characteristics of Spruce Wood Cut by CO_2_ Laser

The final experimental part focused on exploring the optimization of the *v* and *P* technological parameters, which have the most significant effects on all the studied cut characteristics. This part is important because the optimization of the cut region is the main purpose of cutting wood with a laser, and the quality of the cut surface impacts all the studied surface properties, which were the goals of this research, to the greatest extent. This analysis was not the main aim of the paper and represents only additional information. 

The cut quality was optimized according to the following conditions:

- The width of the cut on the bottom and top boards should be minimized;
- The ratio of the cutting kerf widths on both surfaces should be as close as possible to 1.

#### 3.8.1. Cutting Kerf Width Conditions

The cutting kerf widths are presented in Figure 6 and Figure 8. The graphs show that minimal WKU values were obtained with a maximal *v* of 50 mm·s^−1^ and at a *P* of 200 W. Similar results were obtained for the cutting kerf width on the lower board. 

#### 3.8.2. Cutting Kerf Width Ratio Conditions

The cutting kerf width ratios are presented in Figure 10. This graph shows that the WKR was closest to 1 at a maximal *v* of 50 mm·s^−1^ and at a *P* of 200 W.

Both criteria led to the same result: the highest *v* value resulted in the best cut quality. According to the WKR results, the *v* decreased the effect of *P* on the WKR. Considering these aspects, the manufacturing technological process should be focused on changes in *v*, which were proven to have the highest impact on the quality of the cut region.

### 3.9. Sensitivity Analysis of Output Cut Characteristics vs. Input Parameters

Sensitivity analysis is a tool that offers insights into how every single independent input parameter affects the value of the studied output parameter when more than one input parameter affects the results of the output parameter. In general, if a function (*y*) is a function of the *x*1, *x*2, …, *xN* parameters, then the sensitivity coefficient for the input (*xi*) can be computed using Equation (2):

(2) $$\beta \left(xi\right)=xi.\partial y/\partial xi$$

The total deviation of the output parameter (*y*) can then be computed using Equation (3):

(3) $$∆y=\sqrt{\sum_{i=1}^{N}{\beta \left(xi\right)}^{2}}$$

Furthermore, the effect of the parameter (*xi*) on the output (*y*) can be computed using Equation (4):

(4) $$xi\;effect=100\%.\beta \left(xi\right)/∆y$$

Statistica 12 computes the sensitivity coefficients of all the input parameters for the best five neural networks for each output property according to the neural network error. In our study, we assessed the effects of *P*, *v*, and AR on the studied cut characteristic; thus, we obtained a sensitivity coefficient for each input parameter according to Equation (1). The effect of each input parameter on the studied output parameter shown in Figure 6, Figure 7, Figure 8, Figure 9 and Figure 10 was then computed using Equation (3) based on the computation of the total deviation of the output using Equation (2).

The first row is based on data obtained from the measurement, and the second row is based on the prediction data. This routine can be applied if all the statistical parameters of the neural networks for the selected material are very good. Therefore, correlations between 97% and 99% and relative root mean square errors between 1% and 2% for the independent values of the cutting kerf widths and widths of the heat-affected zone are almost at the upper bounds of the possible values for wood, which is inhomogeneous and anisotropic and has an unequal moisture distribution.

We performed a sensitivity analysis of the output cut characteristics versus the input parameters, which provided information on how much changing the input parameters changes the cut characteristics. We performed a sensitivity analysis for every single cut characteristic in two steps. The first step was for data obtained from measurements for *v* = 3, 6, and 9 mm·s^−1^ and *P* = 100 and 150 W, tagged as the measurement subset. The second step was for the *P* and *v*, which were not measured but predicted by neural networks and were thus higher (*v* = 12, 25, and 50 mm·s^−1^ and *P* = 200, 300, 400, and 500 W), tagged as the prediction subset. This analysis also allowed us to assess the effect of a change in the laser parameters on any cut parameter. 

According to Table 1, the *E* values for the measurement subset are lower than those for the prediction subset. Thus, the presented graphs show how the effects of the input parameters change with an increase in the energy density (*E*). The average *E* value for the prediction subset is 43% higher than that for the measurement subset.

#### 3.9.1. Sensitivity Analysis of WKU vs. Input Parameters

**Figure 11:**
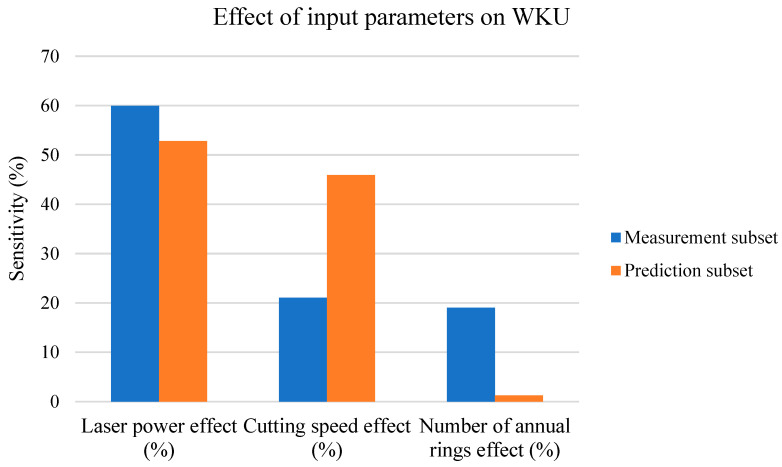
WKU sensitivity analysis.

A sensitivity analysis was performed in Statistica 12.0 software under neural network categorization. In the next paragraphs, we will show the effects of all three input parameters (*P*, *v*, and number of cut ARs) on the measurement and prediction subsets for the WKU parameter, presented in Figure 11 in the form of a column chart.

Figure 11 shows the following:

- The *P* had the strongest effect on the cutting kerf width on the upper board for the measurement subset;
- The effect of *v* on the WKU at *v* values lower than 12 mm·s^−1^ is like that of the number of cut ARs (the second and third blue columns);
- However, increasing the *v* increases the effect of *v* on the WKU by decreasing the effect of *P* and the number of cut ARs (a decrease in the effects for orange columns versus blue columns for both parameters at the same increase as that of the orange column for the *v* effect);
- The effect of the number of ARs on the prediction subset was very low.

The measurement subset is based on the measurement values when the *P* was between 100 and 150 W and the *v* was between 3 and 9 mm·s^−1^ at all AR values from 3 to 11.

The prediction subset is based mainly on *P* values between 200 W and 500 W and *v* values between 9 and 50 mm·s^−1^ at AR values from 3 to 11. These values lead to higher energy densities and, thus, also to a higher amount of heat transfer to the material in the cutting kerf region.

The WKU parameter, according to Figure 2 and Figure 4 (with the measurement subset), increased linearly for the *P* and approx. quadratically for the *v*; thus, both had a strong effect on the WKU. The AR effect on the WKU, according to [10], is linear for the measurement subset; therefore, in the measurement subset, the effects of all three input parameters are statistically significant. The higher *P* effect on the WKU versus the *v* effect is because the cutting speed (*v*) plays a significant role mainly at the lower surface. 

The WKU parameter, according to Figure 6 (the prediction subset), increased with the *P* until it reached the stabilization trend from 300 W. Therefore, for *P* values higher than 300 W, the effect of this parameter on the WKU decreases. According to the same graph, the number of ARs had almost no effect on the WKU, but the increase in the *v* effect on this parameter was the same. Therefore, in the prediction subset, the *v* had a constant effect on the WKU, the *P* had a decreasing effect from *P* values higher than 300 W, and the number of cut ARs had the smallest effect; therefore, in the prediction subset, the effect of *v* on the WKU must be increased at the expense of the *P* and AR effects. 

#### 3.9.2. Sensitivity Analysis of WHAZU vs. Input Parameters

**Figure 12:**
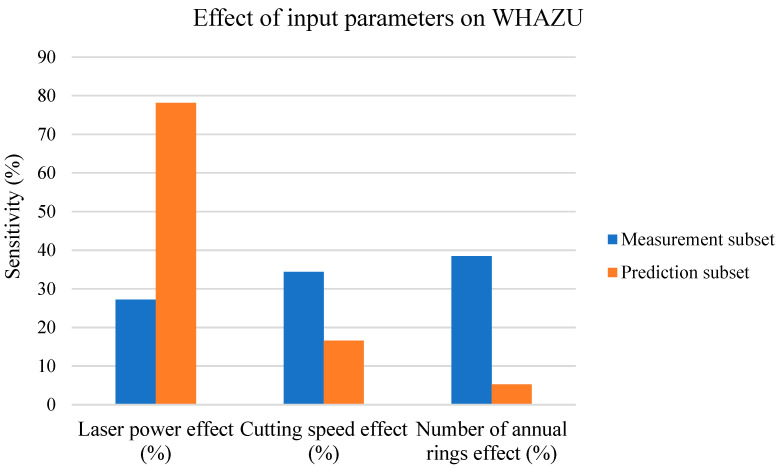
WHAZU sensitivity analysis.

The next paragraphs describe the effects of all three input parameters (*P*, *v*, and number of cut ARs) in the measurement and prediction subsets for the WHAZU parameter, shown in Figure 12 in the form of a column chart.

According to the dependencies shown in Figure 12, the following conclusions can be drawn:

- For the measurement subset, the effects of all three input parameters are approximately the same;
- In the prediction subset, the *P* effect increases at the expense of decreasing *v* and AR effects;
- At higher *P* and *v* values, the number of cut ARs does not play a significant role.

The WHAZU parameter, according to Figure 3 and Figure 5 (with the measurement subset), increased linearly for the *P* and approx. exponentially for the *v*; thus, both had a strong effect on the WHAZU. For the measurement subset, the energy density (*E*) values are lower (Table 1); thus, the AR effect on the studied cut characteristics was non-negligible.

For the prediction subset with higher *E* values, the AR effect on the studied cut characteristics was reduced at the expense of the *P* and *v* effects, leading to a significant decrease in the AR effect on the WHAZU. This is consistent with Figure 7, which shows that the AR effect at higher *v* and *P* values was almost zero; therefore, the AR effect decreased rapidly, as shown in the orange column in Figure 12. Figure 7 also shows that the change in the WHAZU vs. the *v* is approx. 2×–3× lower than the change in the WHAZU vs. the *P*; therefore, at higher *v* and *P* values, the *P* effect increases at the expense of decreasing *v* and AR effects. However, according to the results in Figure 7, none of the changes in the WHAZU in the prediction subset are statistically significant.

#### 3.9.3. Sensitivity Analysis of WKL vs. Input Parameters

**Figure 13:**
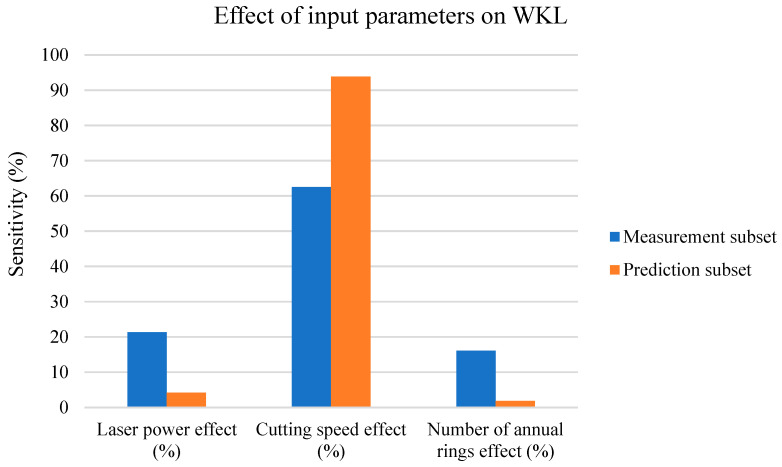
WKL sensitivity analysis.

The next paragraphs discuss the effects of all three input parameters (*P*, *v*, and number of cut ARs) with the measurement and prediction subsets on the WKL parameter, presented in Figure 13 in the form of a column chart.

Figure 13 shows the following:

- The *v* parameter played the most significant role in the cutting kerf width on the lower board;
- For the measurement subset, the AR and *P* effects are approximately the same;
- For the prediction subset, the *v* effect on the WKL increases at the expense of decreasing *P* and AR effects.

The WKL parameter, according to Figure 2 and Figure 4 (with the measurement subset), increased linearly for the *P* and decreased exponentially for the *v*; thus, both had strong effects on the WKL. According to (10), the WKL changes significantly with the AR; therefore, this parameter has approximately the same effect on the WKL as the laser power (*P*). According to these two figures, the decrease in the WKL vs. the *v* was more rapid and intense than the increase with the increasing *P* value; thus, the *v* had the strongest effect on the WKL with the measurement subset (blue columns).

However, according to Figure 8, the AR effect at higher *v* and *P* values was almost zero; therefore, the AR effect decreased rapidly, as shown in the orange column in Figure 12. Figure 8 also shows that the *P* effect on the WKL became less visible after the laser reached a power of 300 W and became higher by increasing the *v* effect on the WKL. Therefore, the *v* effect on the WKL increased for the prediction subset vs. the measurement subset at the expense of reducing the effects of the two other studied parameters (the *P* and AR). 

#### 3.9.4. Sensitivity Analysis of WHAZL vs. Input Parameters

**Figure 14:**
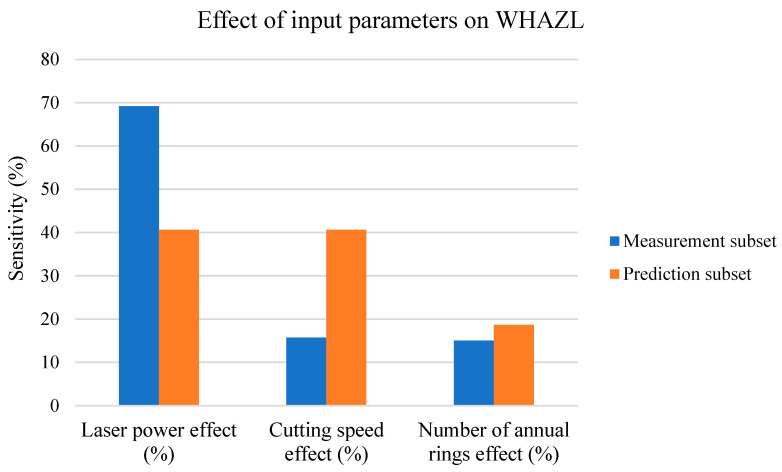
WHAZL sensitivity analysis.

The next paragraphs describe the effects of all three input parameters (*P*, *v*, and number of cut AR) with the training and prediction subsets for the WHAZL parameter, presented in Figure 14 in the form of a column chart.

Figure 14 shows the following:

- For the measurement subset, the effect of *P* on the WHAZL values is greater than the *v* and AR effects;
- The effects of all three input parameters on the measurement subset are significant;
- For the prediction subset, the *P* effect decreases at the expense of an increase in the *v* effect;
- For the prediction subset, the effects of all three parameters are significant.

For the measurement subset, the *v* values are lower, and thus, the creation of the WHAZL is via the heat transfer from the cutting kerf to the heat-affected zone. Figure 3 and Figure 5 show that the effect of *P* on the WHAZL is linear, but the effect of *v* on the WHAZL is stabilizing. The effect of AR is non-negligible because the ARs, through their higher thermal conductivity, increase the WHAZL at the expense of reducing the WKL. However, the creation of the WHAZL is mainly due to the density of the energy transferred to the sample.

As the cutting speed (*v*) increased in the prediction subset, it caused two phenomena: (1) The density of the energy transferred to the sample and, thus, the heat transferred to the sample were lower, and thus, the heat transferred to the WHAZL was also lower. (2) High cutting speeds (*v*) cut the lower surface quicker, which led to the flow of hot air from the cutting kerf to the heat-affected zone, where it increased the smaller WHAZL values. This effect is more significant for higher cutting-speed (*v*) values. These effects then lead to a lower effect of *P* on the WHAZL at the expense of an increase in the effect of *v* on the WHAZL. The effect of AR is not decreased because, when hot air is transferred to the WHAZL, the AR value has a direct effect on the creation of the WHAZL through changes in the thermal conductivity of the wood. This is also consistent with Figure 9, which shows that the effect of *v* on the WHAZL is approximately 2× higher than the effect of *P*. However, the effect of AR is 2× lower, mainly because only *P* and *v* affect the energy density (*E*) values. However, the changes in the WHAZL vs. all three input parameters, according to Figure 9, cannot be considered statistically significant.

#### 3.9.5. Sensitivity Analysis of WKR vs. Input Parameters

**Figure 15:**
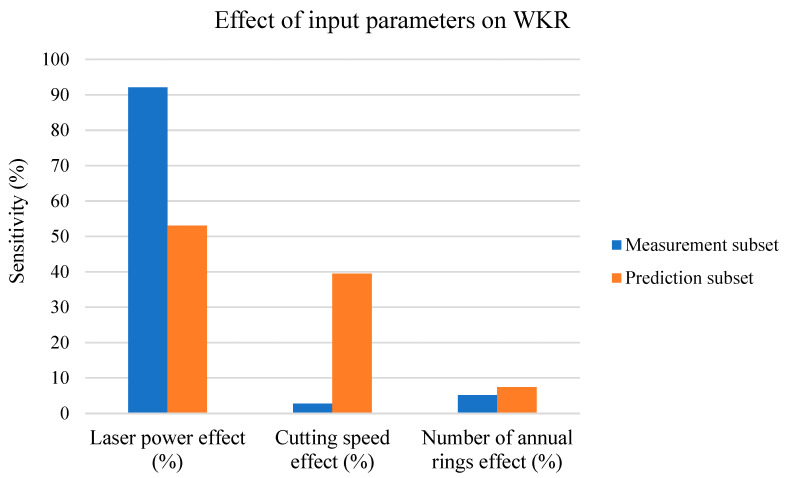
WKR sensitivity analysis.

The next paragraphs discuss the effects of all three input parameters (*P*, *v*, and number of cut ARs) on the measurement and prediction subsets for the WKR parameter, shown in Figure 15 in the form of a column chart.

Figure 15 shows the following:

- For the measurement subset, only the *P* had a significant effect on the WKR value;
- For the prediction subset, the effect of *v* on the WKR increased at the expense of a decrease in the effect of *P*;
- For both the prediction and measurement subsets, the AR on WKR effects are negligible.

The WKR parameter, according to Figure 2 and Figure 4 (with the measurement subset), increased linearly for *P* and did not significantly change with *v*. The AR parameter was mainly affected via changes in the thermal conductivity values of the widths of the heat-affected zones, but the low cutting-speed (*v*) values had no significant effect on the WKU and WKL values, which means that, for the measuring subset, the effect of *P* was very high because neither *v* nor the number of cut ARs had significant effects on the WKR.

However, according to Figure 10, the change in the WKR vs. *P* is negligible after the *P* reaches 300 W, and the change in the WKR vs. *v* is constant (rapidly higher than with the measurement subset), which means that the effect of *P* rapidly decreased compared to the effect of *v*, as shown in Figure 15. In general, the changes in the WKR vs. *v* and *P* are approx. the same, which means that they both had the same effect on the WKR. The small increase in the effect of AR on the WKR (Figure 15) is not statistically significant; therefore, at higher *v* and *P* values, only these two parameters affect the WKR (at an approx. 1:1 ratio).

## 4. Discussion

In this paper, we discuss the effects of all three main technological parameters on the cutting kerf properties of spruce wood. Former studies dealt with the prediction of these parameters with constant *v* values, which is applicable only for certain cutting speeds.

The other laser parameters were not changed because their effects on the studied final cut characteristics are, according to the references, not as strong as the effects of *P* and *v*. The parameters that significantly affect the cut characteristics are based on the microstructure of the material. These parameters can be recorded only before cutting and then only the final cut results are recorded, and they cannot be inspected during the cutting process; thus, their effects cannot be measured or quantified. 

When predicting the cut parameters vs. all the changing technological properties, these predictions cover all possibilities. Therefore, this process can be successfully applied to any spruce wood cut with a CO_2_ laser with *P* values between 100 and 500 W, all possible combinations of the number of cut ARs (i.e., any structure of spruce wood), and all possible used *v* values between 3 and 50 mm·s^−1^, based on just on two combinations of *P*, three combinations of *v*, and nine combinations of the number of cut ARs, which reduces the material and energy consumption. The presented results should be taken into consideration as qualitative trends; thus, the values of the cutting kerf parameters can change, but the best combination of technological parameters is the same for all types of spruce wood cut with a CO_2_ laser with *P* values between 100 and 500 W and *v* values between 3 and 50 mm·s^−1^.

The sensitivity analysis added to this study offered a quantitative look at the trends of all the studied cut characteristics vs. the input values, which added numerical information to the trends, providing qualitative results. The sensitivity coefficients can be regarded as constant for selected types of wood in the regions of the input values, which were measured or predicted. Moreover, the analysis also allowed us to focus the research on the optimal *v* and *P* values for the best possible cut quality by lowering the amount of input parameter combination testing. This analysis helps to determine which input parameters at which energy density values have the most significant effect on each one of the studied cut parameters.

## 5. Conclusions

In the presented research, we focused on the effects of laser power (*P*), cutting speed (*v*), and number-of-cut-AR on the studied cut characteristics of spruce wood performed with a CO_2_ laser for all possible values at which this material can be cut by a CO_2_ laser. The main conclusions are as follows:

- Based on the measured values of the cut characteristics versus the input values, it is possible to predict the cut characteristics vs. the input values for all possible values at which spruce wood can be cut by a CO_2_ laser;
- The effects of the chosen input parameters on the studied cut characteristics were mainly dependent on the density of the energy (*E*) transferred to the sample and the cutting speed (*v*);
- The effect of AR is significant only for low *E* values;
- With higher *E* values, the effect of AR is negligible;
- The cutting speed (*v*) has a significant effect mainly on the cut characteristics of the lower board;
- The laser power (*P*) has a more significant effect on the cut characteristics of the upper surface (the side on which the laser beam is initially applied);
- The WHAZU (WHAZL) values increase at the expense of reduced the WKU (WKL) values, mainly because of the heat transfer from the cutting kerf to the heat-affected zone;
- The WHAZL values are significantly higher than the WHAZU values, which is because of the reduction in the WKL values because of the heat transfer from the cutting kerf to the heat-affected zone;
- The effect of *P* on the WKU (WKL) stabilizes after reaching a laser power (*P*) equal to 300 W, which then leads to an increase in the effect of *v* on both the WKU and WKL and, thus, also the WKR.

From the presented results, the following can also be concluded:

- The best cut quality of spruce wood can be obtained with a maximal cutting speed (*v*) value of 50 mm∙s^−1^, where the WKU and WKL values are almost the same for the laser power (*P*) and approximately 250 W for any number-of-cut-AR value;
- The sensitivity analysis showed the effects of *P*, *v*, AR on the cut characteristic change versus the value of the density of the energy (*E*) applied to the upper surface;
- The AR effect decreased at higher energy densities (*E*) because this parameter does not affect the amount of energy transferred to the material;
- For the majority of the studied cut characteristics (except the WHAZU), the effect of *v* increased with an increase in the energy density (*E*) value.

## Figures and Tables

**Table 1:** Energy density (*E*) values vs. laser power (*P*) and cutting speed (*v*).

Power (*P*) [W]		100	150	200	300	400	500
	Energy Density (*E*) [J∙mm^−2^]						
**Cutting speed** **(*v*) [mm·s^−1^]**	3	111	167	222	333	444	556
	6	56	83	111	167	222	278
	9	37	56	74	111	148	185
	12	28	42	56	83	111	139
	25	13	20	27	40	53	67
	50	7	10	13	20	27	33

**Table 2:** Nonlinear mathematical regression statistical parameters.

Property	WKU (10^−3^ m)	WKL (10^−3^ m)	WKR (-)	WHAZU (10^−3^ m)	WHAZL (10^−3^ m)
R^2^ (-)	0.86	0.88	0.63	0.57	0.51

**Table 3:** ANN statistical parameters.

Property	WKU (10^−3^ m)	WKL (10^−3^ m)	WKR (-)	WHAZU (10^−3^ m)	WHAZL (10^−3^ m)
R^2^ (-)	0.95	0.98	0.93	0.96	0.97

## Data Availability

Data are contained within the article.
